# Lissencephaly with Congenital Hypothyroidism: A Case Report

**DOI:** 10.31729/jnma.7893

**Published:** 2022-11-30

**Authors:** Shambhu Kumar Sahani, Anil Pathak, Bishal Nepali, Nilshan Rai

**Affiliations:** 1 KIST Medical College and Teaching Hospital, Imadol, Lalitpur, Nepal

**Keywords:** *congenital abnormalities*, *hypothyroidism*, *lissencephaly*, *neuronal migration disorders*

## Abstract

Lissencephaly is a malformation of cortical development associated with deficient neuronal migration and abnormal formation of cerebral convolutions or gyri. The lissencephaly spectrum consists of agyria, pachygyria, and subcortical band heterotopia. At least 19 genes have been identified in the causation of lissencephaly and related syndrome. Lissencephaly is associated with many other congenital disorders but the association of lissencephaly with congenital hypothyroidism is rarely reported. We report a case of a 10-year-old girl having lissencephaly with congenital hypothyroidism. Early diagnosis of lissencephaly and genetic counselling can be made in suspected cases and further possible interventions can be taken. Also, regular follow-up, monitoring, and better conservative management lead to a better prognosis.

## INTRODUCTION

Lissencephaly, literally "smooth brain", is a condition characterised by decreased gyral and sulcal development of the cerebral surface.^1^ It is due to the defective neuronal migration prenatal period (that is in between 12 and 24 weeks of gestation), and there is an abnormally thick cortex and absent or reduced formation of cerebral convolutions.^2,3^ Lissencephaly is divided into main two large groups: Classical lissencephaly and cobblestone lissencephaly.^2^ More than 90% of the individual with lissencephaly has a seizure and also there is mild to severe developmental delay, poor feeding, mild to moderate hypotonia, and poor visual and sound response.^1^ There is no specific treatment for this condition and it is managed symptomatically.^4^

## CASE REPORT

**Figure 1:**
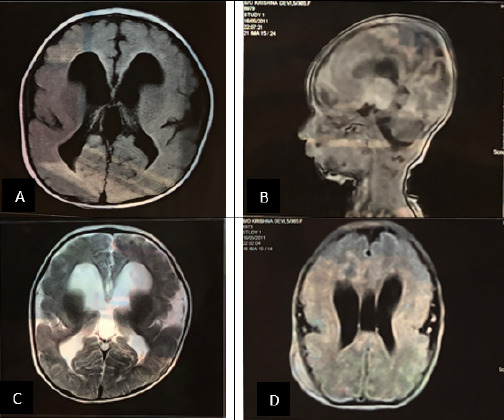
MRI showing ventriculomegaly with smooth parenchyma (A: T1 axial view, B: sagittal view, C: T2 axial, reduced sulci and gyri with smoothness, D: Axial/flair with cavum septum pellucidum/vergae).

A 10 years 11 months female child was born at 37+4 weeks of gestation via vacuum-assisted vaginal delivery without requiring any neonatal resuscitation, a product of non-consanguineous parents from a chronic hypertensive mother. Mother was on antihypertensive medications. On antenatal screening abdominal USG (at 35 weeks of gestation) showed agenesis of the corpus callosum, mild ventriculomegaly, and nonvisual cavum septum pellucidum. On the 3-4^th^ day of life, the baby developed jaundice but didn't require phototherapy. On the postnatal 5^th^ day of life an MRI was done which showed defects in neuronal migration and subgaleal hematoma and on the 15^th^ day of life diagnosed to have hypothyroidism for the first time on a routine thyroid function test (Figure 1).

**Figure 2:**
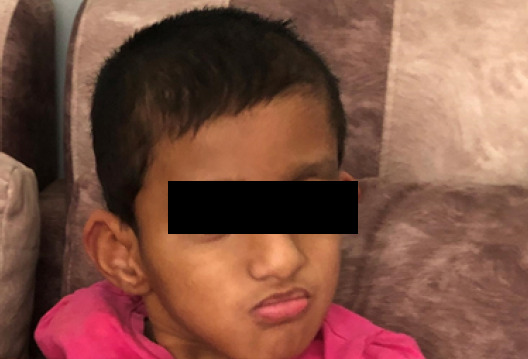
Facial features show microcephaly, enlarged ears, and broad nasal bridges.

At 2 years of life, the child developed abnormal movement of the body and was given sodium valproate. At 11 years of age, she was unable to speak but could respond to sound, had muscle in-coordination, and couldn't stand by herself, and had very little interaction with children of the same age group. All these features were suggestive of global developmental delay. On examination, there were microcephaly, enlarged ears, broad nasal bridges, visible chest retraction (pectus excavatum), generalised muscle weakness, and hypotonia (Figure 2).

Family history showed congenital heart disease in his 18 years old brother as well. On her recent hospital visit at 10 years of age, she was diagnosed with right level II and III lymphadenopathy and collection anterior to the body of the right mandible. She had multiple dental caries and intraoral abscesses for which conservative management, full mouth extraction, and full mouth rehabilitation were done. Anthropometric assessment at age of 10 years showed length 117 cm, weight 19 kg, mid-upper arm circumference 15 cm, and head circumference 49 cm. Her different growth parameter are: weight for age, Z-score= -4.03 (severely underweight), Height for age, Z-score= -3.61 (severely stunted), Weight for height, Z-score= -1.23 (normal parameter, no wasting), BMI=13.9 [according to WHO, CDC growth chart], Head circumference Z-score= -3.39 (microcephaly) [according to Kromeyer-H data]. Thyroid function test (TFT) at 9 months showed U-TSH;29.0 UI/ml, free T3;2.5 pg/ml, and free T4;0.7 ng/dl which signifies congenital hypothyroidism for which she is under medication of thyroxine 50 mcg (Table 1).

**Table 1:** Thyroid Function Test.

Date	12/02/068 (at 15^th^ days of life)	23/10/068 (at 9 months)	17/10/077 (at 9 years)	05/11/077 (at 9 years)	25/11/077 (at 9 years)	19/08/2078 (at 10 years)
U-TSH (uUI/ml)	26.0	29.0	>150	3.12	0.66	5.82
FREE T3(pg/ml)	1.00	2.5	1.35	5.93	7.55	3.39
FREE T4(ng/ml)	0.46	0.7	0.40	2.39	3.22	1.78

At 9 years immunological test for anti-thyroid-specific peroxidase showed 122 IU/ml (Normal: <34). At 24^th^ days of life colour doppler echocardiography showed situs solitus, levocardia, D-loop ventricles with normal segmental analysis, OS ASD 7 mm in size, left to right shunt, perimembranous VSD, 7 mm in size, left to right shunt with normal left ventricular function. Trans VSD GRAD 41 mm Hg s/o elevated PA pressure. At 11 months sound field assessment by distraction/visual reinforcement technique showed severe hearing impairment (stimulus presented to both right and left side is 70 dB on all high, mid, and low frequency). Audiometry brainstem response (ABR) done at 9 months showed moderate hearing impairment. On the investigation done at 10 years, MRI showed:

- Gross hydrocephalus of frontal horns and body of bilateral lateral ventricles and 3^rd^ ventricle and mild dilatation of the bilateral temporal and occipital horns and 4^th^ ventricle without significant periventricular ooze.
- Diffusely thinned out the white matter with a few small T2/FLAIR hyperintensities in the subcortical and periventricular white matter of bilateral frontal lobes.
- Thick cortex in bilateral frontoparietal regions with sparse cortical sulci at these regions.
- Prominence/widening of extra-axial CSF spaces underlying bilateral frontal and temporal convexities, bilateral System Sylvian fissures, & suprasellar, bilateral ambient, prepontine, and bilateral CP angle cisterns.
- Few small T2/FLAIR hyperintensities, subcortical and periventricular white matter of bilateral frontal lobes.
- Cavum Septum Pellucidum and Cavum Vergae.

The above features may represent hydrocephalus with pachygyria complex (Lissencephaly). Mucosal thickening of Right maxillary sinus. In this case, we don't have any genetic testing rather diagnosis was made based on a radiological and clinical basis. Symptomatic management was done as follows: Earlier since birth, she was on regular medication of levothyroxine 50 mcg but for the last 3 months is taking levothyroxine 100 mcg once daily empty stomach before breakfast and after food, she is taking levetiracetam twice daily but on morning dose is 250 mcg and the evening dose is 500 mg, and lacosamide 50 mg once daily at bedtime (since 1.5 years) for her seizure control.

## DISCUSSION

Lissencephaly means "smooth brain," which is a rare, gene-associated brain malformation characterised by the absence of normal sulci and gyri in the cerebral cortex. It is due to neuronal migration defects. Recent advancement in radiological imaging technique has led to the identification of various patterns of lissencephaly and its associated syndromes. The various neuronal migration disorders are agyria, mixed agyria/pachygyria, polymicrogyria, abnormally thick and poorly organised cortex with primitive layers, lissencephaly, diffuse neuronal heterotopias, enlarged and dysmorphic ventricles, often hypoplasia of corpus callosum, teratomas, and phakomatosis.^5^ The exact prevalence of lissencephaly is not known though it is estimated to range from 11.7 to 40 per million.^6^

The spectrum of the symptoms of the disorder may include seizures, severe psychomotor retardation, unusual facial appearance, difficulty swallowing, failure to thrive, and muscle spasms. Hands, fingers, or toes may be deformed.^4^ In our case, there are seizures, failure to thrive, severe psychomotor retardation, and difficulty feeding. Various forms of Classical lissencephaly (also called type I lissencephaly) include anomalies in the LIS1 gene (isolated lissencephaly and Miller-Dieker syndrome (MDS), anomalies in the TUBA3 and DCX genes, and lissencephaly caused by mutations in the ARX gene (XLAG syndrome, X-linked lissencephaly with agenesis of the corpus callosum). Cobblestone lissencephaly (also called type II lissencephaly) is present in 3 forms as Walker-Warburg, Fukuyama, and MEB (Muscle-Eye-Brain) syndromes.^2^ Lissencephaly with facial dysmorphisms is seen in MDS, Baraitser-Winter syndrome (BWS), and NormanRobert syndrome.^3,5^ Features like microcephaly, high forehead, bitemporal hollowing, vertical furrowing of the forehead when crying, flattened ear helices, mild hypertelorism, the broad nasal bridge with epicanthal folds, anteverted nares, prominent lateral nasal folds, a flat midface, and a small chin is seen in MDS.^5^ Genetic tools were not used in our case and the diagnosis was made on the basis of radiological(shown in figure 1) and clinical findings. Based on the findings, our case suggests being Type 1 lissencephaly. Clinically Prognosis of this condition is very poor and is about 10 years, death usually occurs due to aspiration of foods, respiratory disease, and severe seizures.^4^

At least 19 genes have been involved in the causation of lissencephaly, most of which are related to the microtubule structural protein of microtubule-associated protein. These lissencephaly-related genes include LIS1, DCX, ACTB, ACTG1, ARX, CDK5, CRADD, DYNC1H1, KIF2A, KIF5C, NDE1/NDEL1, TUBA1A, TUBA8, TUBB, TUBB2B, TUBB3, TUBG1, RELN and VLDLR.^3^ Lis1 (also called PAFAH1B1) and DCX are the common and first identified genes involved in lissencephaly. Three genes cause type1 lissencephaly: LIS1, TUBA1A, and DCX(also called doublecortin).^6^ LIS1 gene is located on chromosome 17p13.^3^ and encodes for platelet-activating factor acetylhydrolase isoform 1B which interacts with microtubule-associated proteins dynein and dynactin. This interaction is critical for proper neuronal migration during foetal brain development and disruption of this interaction results in lissencephaly.^7^ DCX and ARX-associated lissencephaly are called X-linked lissencephaly type 1 and 2, respectively (XLIS 1-2 or LISX 1-2). DCX and ARX are genes located on the X chromosome, and their mutation in males (in a hemizygous state) causes lissencephaly.^6^ Females having this gene mutation present with a milder form of lissencephaly.^7^ Ambiguous genitalia and absent corpus callosum are associated with X-linked lissencephaly. Mutation of the reelin(RELN) gene causes autosomal recessive lissencephaly with cerebellar hypoplasia.^3^ Mutations in the tubulin a-1A (TUBA1A) gene are known to be associated with a distinctive radiologic phenotype consisting of posteriorly predominant lissencephaly with corpus callosal dysgenesis, cerebellar and brainstem hypoplasia, and polymicrogyria.^8^ In half of the patients having Cobblestone lissencephaly(type II lissencephaly), there is a mutation in POMT^1^, POMT^2^, POMGNT^1^, FKTN, FKRP, and LARGE genes. In this condition, there is over a migration of neurons as compared to under migration of neurons in classical lissencephaly.^6^

Lissencephaly may be due to various non-genetic and genetic factors and these factors include intrauterine infection, an insufficient supply of oxygenated blood to the brain (ischemia) during foetal development, and/or different gene mutations.^5^ For normal brain development action of thyroid hormone is critical and is mediated by both nuclear and extranuclear pathways.^9^ According to an experimental study, developmental iodine deficiency and hypothyroidism impair the expression of doublecortin and NCAM-180, leading to nerve fibre malfunction, and impairment in hippocampal development.^10^ Direct association between regulation of reelin (RELN) and dab1 gene and thyroid hormone has been found. In the animal model, it has been shown that hypothyroidism cause defect in the expression of reelin and dab^1^ gene thus neuronal migration and lamination during brain development.^11^ Studies have found that mutation in the MCT8 (SLC16A2) gene, thyroid hormone coding gene caused severe psychomotor retardation, severe neural impairment, low serum thyroid level as well structural brain abnormalities.^12,13^ These associations show that there may be a relation between hypothyroidism and the causation of lissencephaly. In that case, there is a four-day-old male who had features of lissencephaly, diagnosed based on radiology, with congenital hypothyroidism.^5^

Till now, the aetiology of lissencephaly is poorly understood especially in the case of association of lissencephaly with congenital hypothyroidism and we reported this case to show if there is any probable relationship between Lissencephaly and hypothyroidism. Several studies have been carried out that have shown the association between normal central nervous system development and optimum thyroid hormone level. More studies need to be conducted to confirm the association and to find out the possible effective intervention. Early diagnosis of lissencephaly and genetic counselling can be done in suspected cases and further possible interventions can be taken. Also, regular follow-up, monitoring, and better conservative management lead to a better prognosis as seen in our case as well.
